# Validation of a Patient-Completed Caprini Risk Score for Venous Thromboembolism Risk Assessment

**DOI:** 10.1055/s-0037-1607339

**Published:** 2017-10-20

**Authors:** H. E. Fuentes, L. H. Paz, A. Al-Ogaili, X. A. Andrade, D. M. Oramas, J. P. Salazar-Adum, L. Diaz-Quintero, C. Acob, A. Tafur, J. Caprini

**Affiliations:** 1 Department of Medicine, Division of Internal Medicine, John Stroger Jr. Hospital, Chicago, Illinois, United States; 2 Department of Pathology, University of Illinois at Chicago, Chicago, Illinois, United States; 3 Department of Medicine, Division of Internal Medicine, NorthShore University HealthSystem, Evanston, Illinois, United States; 4 Department of Medicine, Division of Cardiology and Vascular Medicine, NorthShore University HealthSystem, Evanston, Illinois, United States; 5 NorthShore University HealthSystem–Emeritus, Pritzker School of Medicine, Evanston, Illinois, United States

**Keywords:** Caprini score, venous thromboembolism, risk assessment model, thrombosis prophylaxis, patient-centered communication

## Abstract

**Introduction** Individualized risk assessment for venous thromboembolism (VTE) using the Caprini risk score (CRS), coupled with targeted prophylaxis based on the score, is effective in reducing postoperative VTE. Critics contend that using this tool is time consuming for health care providers. We decided to create a patient-completed CRS and conducted a prospective study to compare the scores calculated by a patient with those calculated by a blinded physician for the same patient.

**Methods** In phase 1, we interviewed patients in our deep vein thrombosis (DVT) support group who had a history of thrombosis and included their family members to determine areas of misunderstanding in the original CRS. We created a patient-completed form based on these interviews. In phase 2, we further optimized the questions after a CRS-trained, blinded physician scored 20 hospitalized patients during the pilot study. In the final (third) phase, we measured the agreement level between the new form filled out by the trained physicians and those filled out by the patients. The study was approved by our local institutional review board. Using PASS version 11, we determined that a sample size of 37 individuals achieves a power of 80%, to detect a 0.1 difference between the null hypothesis correlation of 0.5 and the alternative hypothesis correlation of 0.7 using a two-sided hypothesis test with a significance level of 0.05. We tabulated the individuals' answers and categorized the scores by using SPSS version 23 to estimate the kappa value, linear correlation, and the Bland–Altman test. A kappa value greater than 0.8 indicated an “almost perfect agreement.”

**Results** We tested the first patient-completed CRS version (phase 2) in a 20-patient pilot study. A poor agreement was observed with the body mass index (BMI) responses in multiple iterations, and so we excluded the BMI calculation from the final patient-completed CRS form. We recruited 42 patients with an average age of 55, mostly female (45%), who completed less than college education (62%) to fill out the updated CRS form (phase 3). An almost perfect agreement was found for both the individual questions and the overall score comparing physician and patient answers, resulting in a high correlation ( *r* = 0.95). In Bland–Altman, we did not find any trend for extreme values.

**Conclusion** We created and validated a patient-completed CRS form that has an excellent agreement level with the physician-completed form. From the results, the physician only needs to calculate the BMI. The average time for a patient to complete the form was 5 minutes. The average time for the physician to finalize the score was approximately 6 minutes. Implementation studies are needed to assess the correlation of the aggregated score, derived from this form, with the occurrence of perioperative VTE.

## Introduction

Perioperative venous thromboembolism (VTE) is a leading preventable cause of morbidity and mortality in surgical patients. 1 It represents the second most common postoperative complication and the third most common cause of excess mortality and cost in surgical patients. 2 Primary thrombosis prophylaxis is an effective and safe strategy to reduce VTE-related complications. 3 The Agency for Healthcare Research and Quality identified VTE prevention as the most important strategy for improving the safety of hospitalized patients. 4

The timing and necessity for primary VTE prophylaxis are decisions that are based on balancing both patient- and procedure-specific risks of bleeding and thrombosis. The 2005 Caprini risk score (CRS) weighs individual risk factors to create an aggregate risk score that correlates with postoperative VTE in nonorthopaedic surgical patients. The Caprini model consists of 38 individual risk factors weighted from 1 to 5 points depending on the likelihood of an individual factor to be associated with the development of VTE. 5 The score has been extensively validated in multiple surgical subspecialties. 6 7 8 9 10 11 The implementation of an individualized VTE prevention strategy based on the original health care provider–completed CRS has lowered the incidence of postoperative VTE. 12 The American College of Chest Physicians (ACCP 9th edition) recommends the use of the CRS as one method for the risk assessment of nonorthopaedic surgical patients. 12 Critics of the CRS contend that using the tool is time consuming for health care providers 13 which potentially limits its applicability.

A growing body of evidence suggests that patients and their family members want more education regarding prevention, significance, and consequences of VTE. 14 We created an online patient-completed version of the Caprini model to facilitate calculation of the Caprini score. 15 We are unaware of the correlation between the aggregated score calculated by the physician and that calculated by patients with the use of this on-line tool. Formal evaluation of these two methods has not been previously done. This study was undertaken to address this concern and assess the advantages of patient-centered communication regarding VTE. 16 17 18 We decided to create and validate a patient-completed version of the CRS, as no patient-completed validated instruments are currently available.

## Methods

### Patients and Methods

The validation process was divided into three phases.

### Phase 1

We held a focus group from members of a local deep vein thrombosis (DVT) support group. Patients who had a history of VTE and their family members filled out the existing CRS form (Phase 1). The aim of this exercise was to determine areas of misunderstanding in the original CRS. One of five trained physicians completed the original CRS version on the same patients and transcribed their observations. We used a deductive approach to analyze both sets of responses until data saturation was reached. 19 On the basis of these interviews, a patient-completed CRS form was created. This instrument was tested in hospitalized patients in phases 2 and 3.

### Phase 2

We prospectively recruited patients admitted to the John Stroger Jr. Hospital from October 2016 through January 2017. We included patients older than 18 years admitted to a medical or surgical unit. We excluded illiterate patients and those with altered mental status, visual disorders, and acquired/congenital cognition impairment.

**Fig. 1:**
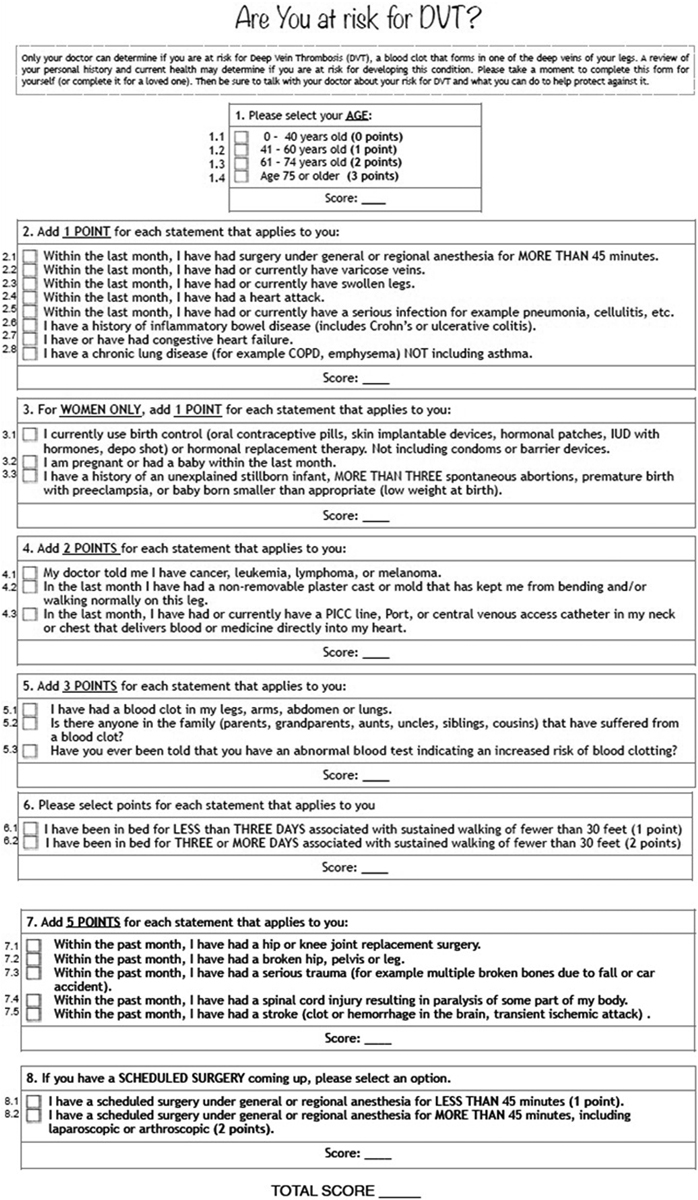
Patient-completed Caprini risk score form.

We conducted a pilot study on 20 patients to refine the CRS document (Phase 2). In an interim analysis, we measured the level of agreement between each item of the patient- and the physician-completed CRS to specifically target the problem questions. During the first part of the interview, the patients calculated their patient-completed CRS with no assistance or instructions other than those in the form. Subsequently, a physician scored the CRS for the same patient on the basis of information acquired from a formal patient exam and electronic medical records (EMR). The scoring physician was blinded to the patient's report, and the scores for both patient and physician were compared. Basing on the results of this comparison and the patients' suggestions, we produced the final form ( Fig. 1 ).

### Phase 3

During a 4-week period, we prospectively enrolled patients admitted to the medical and surgical units for the first 48 to 96 hours of admission. The patients completed the form and this was compared with the blinded physician interview. Each physician interviewer underwent a training session on the CRS by one of the senior authors (A.T.) before the validation process was commenced.

The institutional review board approved this study and waived signed consent.

### Statistical Analysis

We calculated Cohen's kappa (k) to quantify the question-to-question agreement between the patient-completed CRS and the physician-completed CRS. The Bland–Altman method was used to evaluate the agreement between the scores calculated by the patients and those by the physician. 20 We computed the Spearman's correlation coefficient to assess the construct validity and correlation of the overall scores. Finally, we categorized the overall CRS based on ACCP guidelines 12 into very low (0), low (1–2), moderate (3–4), and high risk (≥5), and we measured the agreement level by using kappa. Kappa values of 0.4 or less are considered as poor, 0.41 to 0.60 as moderate, 0.61 to 0.80 as substantial (good), and 0.81 to 1 as almost perfect (excellent) agreement. 21 Using Power Analysis and Sample Size Software (PASS) version 15 (NCSS, LLC, Kaysville, Utah, United States), we determined that a sample size of 37 achieves a power of 80%, to detect 0.1 difference between the null hypothesis correlation of 0.5 and the alternative hypothesis correlation of 0.7 using a two-sided hypothesis test with a significance level of 0.05. Categorical and continuous data are presented as percentages and median range. All statistical analyses were conducted in SPSS version 22 (IBM Corp., Armonk, New York, United States)

## Results

There were five focus-group sessions with eight participants each in phase 1. The main areas of concern were the presence of the item “age” in multiple sections of the original form, the concept of BMI, the concept of lung disease, and identification of chronology of events in the statement “now or within the past month” in the original form. The form created for the pilot addressed those questions and used multiple strategies to answer more challenging topics, including BMI. We used this form during phase 2 of the study.

In phase 2 of this study, we recognized consistently poor agreement ( *k* < 0.4) in three major areas of the initial form: chronology of prior medical history, interpretation of mobility and ambulation, and accuracy of the BMI. We tested the modifications in the final validation phase. 3 For this, we enrolled 42 patients with an average age of 50 years (median: 55; range: 19–96), 23 (54.76%) were men with less than college education (62%). The median CRS calculated by the physician was 6 (range: 0–16) and the majority (64.3%) were classified as high risk for VTE based on the CRS ( Table 1 ). Patients spent a median time of 5 minutes (range: 3–7) completing the form. Physicians completed the verification process in approximately 6 minutes.

**Table 1:** Patient characteristics and median Caprini risk score

Variables	Cohort, *n* = 42
Age (mean [range])	55 (19–96)
Gender (%)	
Women	19 (45.2)
Men	23 (54.8)
Education level (%)	
Elementary	5 (11.9)
High school	21 (50)
College	13 (31)
Postgraduate	3 (7.1)
Patient-completed score (median [IQR])	6 (4–9)
Physician-completed score (median [IQR])	6 (4–8)

Abbreviation: IQR, interquartile range.

The problems found in phase 2 were addressed. First, to clarify chronology of events, we added “within the last month” at the beginning of each sentence. This modification yielded a substantial to almost perfect agreement for the applicable items in the final phase ( Table 2 ).

**Table 2:** Agreement level questions-to-questions

Item	Kappa
1.1	1.000
1.2	1.000
1.3	1.000
1.4	1.000
2.1	0.940
2.2	1.000
2.3	1.000
2.4	1.000
2.5	0.896
2.6	1.000
2.7	1.000
2.8	1.000
3.1	1.000
3.2	1.000
3.3	1.000
4.1	0.940
4.2	1.000
4.3	0.846
5.1	1.000
5.2	0.844
5.3	1.000
6.1	0.877
6.2	0.893
7.1	1.000
7.2	1.000
7.3	1.000
7.4	1.000
7.5	1.000
8.1	0.844
8.2	0.844

Note: Shows agreement level for each item of the patient-completed Caprini risk score.

Second, the immobility definition in the original CRS was adopted from the Medical Patients with Enoxaparin (MEDENOX) trial; 22 thus, we explicitly asked about their walking distance. This implementation resulted in a *k* : 0.9 for both questions addressing immobility ( *k* : 0.9, 95% CI: 0.83–0.96; *k* : 0.9, 95% CI: 0.80–96).

Third, BMI was not accurately calculated based on the information provided by patients in any of the facilitated question iterations. We inquired about the BMI in three different ways: (1) by asking patient their height and weight, (2) providing a chart to calculate the points adjudicated by the CRS, based on their height and weight, and (3) by directly asking their BMI. Therefore, we decided by consensus to remove BMI from the patient-completed CRS in subsequent analyses, and concluded that it should be added in the final intake by the prescribing physician.

**Fig. 2:**
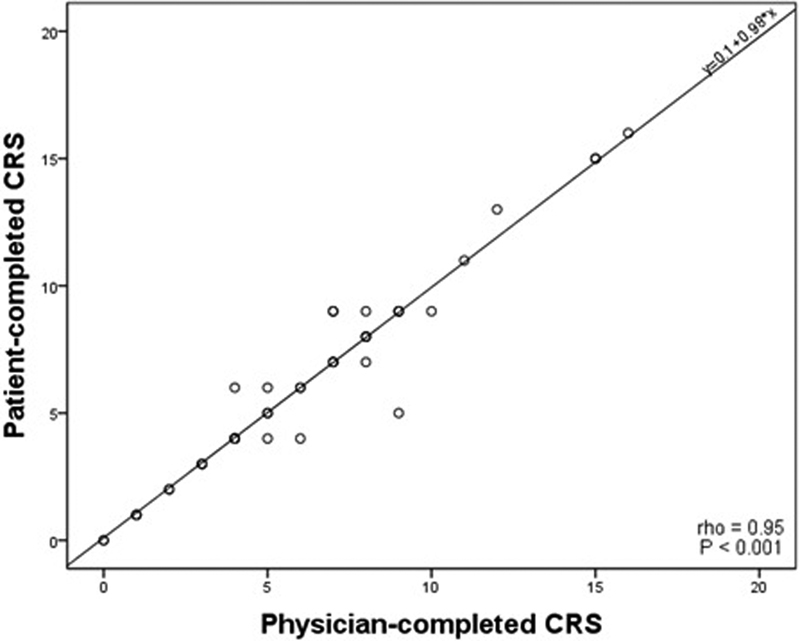
Correlation for patient-completed Caprini risk score.

**Fig. 3:**
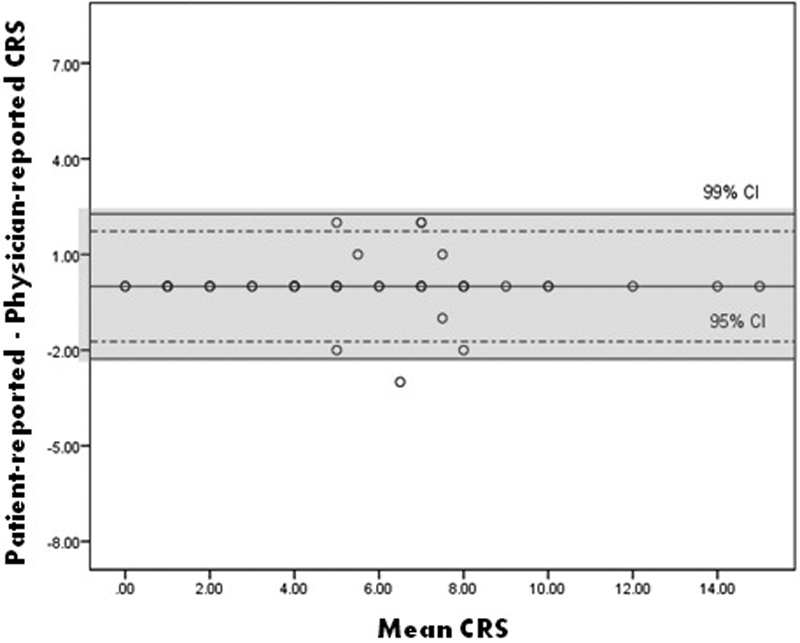
Bland–Altman for patient-completed Caprini risk score.

The agreement for the cumulative CRS score was 0.9 (95% CI: 0.73–1.00) when CRS was categorized following ACCP guideline recommendations. Because higher levels of postoperative VTE are seen with CRS levels of 8 and 11, we further divided the CRS using these cutoff values. 6 23 24 The analysis resulted in a *k* : 0.8 (95% CI: 0.65–0.85) and *k* : 1.00 (standard deviation: 0.00) for the second cutoff value ( Table 3 ). Spearman's correlation coefficient between patient- and physician-completed forms was 0.95 ( *p* < 0.01; Fig. 2 ). We did not find any trend for the extreme values using the Bland–Altman method ( Fig. 3 ).

**Table 3:** Agreement level by stratification

CRS stratification	Kappa
ACCP stratification	0.9
CRS above 8	0.8
CRS above 11	1.000

## Discussion

We created and validated the first patient-completed version of the most widely used perioperative VTE risk assessment model, which showed excellent correlation. This English form may facilitate the use of the Caprini score to assess nonorthopaedic surgical patients' thrombosis risk in the United States and in other countries where English is used. Providing a clear-cut estimate of thrombosis risk may aid physicians in providing appropriate thrombosis prophylaxis that will reduce the incidence of perioperative symptomatic VTE. Additional evaluation of patient-friendly forms in other languages is underway using the methods in the current study.

The concept of scoring VTE risk has led to multiple VTE risk assessment tools including the Kucher score, 25 the Padua score, 26 the Rogers score, 27 the Caprini score, 5 and recently the improved score. The 2005 Caprini score is the most extensively validated instrument and is widely endorsed by societies, organizations, and guideline documents for assessing VTE risk in surgical patients.

One of the most successful programs using the score in surgical patients was done by Cassidy and associates in a hospital-wide program using an EMR-based CRS. 28 The scores were used to dictate the nature and duration of VTE prophylaxis including an outpatient component. The score was calculated at the time of admission or preoperatively in every surgical patient. The results were linked to electronic prophylaxis recommendations which had to be acted upon before the orders could be signed. The prophylaxis regimens provide the recommended mechanical and pharmacologic prophylaxis along with the recommended duration of chemoprophylaxis. Patients with a risk score of 0 to 4 received prophylaxis during hospitalization and many patients with lower scores were prescribed mechanical methods alone. The results of the program have shown that these patients can be spared anticoagulation prophylaxis without sacrificing efficacy and thus avoid bleeding complications.

The unique feature of the Cassidy regimen was prescribing extended prophylaxis for 7 to 10 days for scores from 5 to 8, and 30 days of low-molecular-weight heparin prophylaxis for those with scores of 9 or greater. There is a drop-down menu that indicates reasons for not prescribing chemoprophylaxis such as an increased bleeding risk. During the audit period, 100% compliance was achieved in the low- and moderate-risk groups and 89% compliance in those who were at high risk. The 30-day clinically proven PE rate during the audit period decreased from 1.1 to 0.5%. 28 A vigorous program of ambulation was also incorporated into the postoperative orders.

A recent a meta-analysis involving 14,776 patients in 13 eligible studies examining VTE events was conducted. Eight studies in 7,590 patients contained data for clinically relevant bleeding with and without anticoagulant prophylaxis. These studies examined the benefit of postoperative chemoprophylaxis according to Caprini's scores. The majority of patients received mechanical prophylaxis. VTE risk in patients who did not receive chemoprophylaxis varied between 0.7 and 10.7% according to the Caprini model. Those with higher scores were significantly more likely to suffer VTE events. Patients with scores of 6 or less comprised 75% of the overall 14,776 patients. These patients did not have significant reduction of VTE using chemoprophylaxis. Significant benefit using chemoprophylaxis was seen in those with scores of 7 or more. 29 This meta-analysis showed that many patients can be spared anticoagulant prophylaxis without sacrificing efficacy. The cutoff points between these studies vary slightly which may reflect differences in individual populations—one being a single center and the other representing a variety of sites using data from multiple countries around the world.

Although robust evidence supports the efficacy of the Caprini model in identifying surgical patients at a high risk for postoperative VTE, including in the posthospitalization period, 6 23 critics contend that the use of this tool is time consuming for health care providers. 13 Nonetheless, in our study the patients required 5 minutes, on average, to complete the form which had an excellent correlation with the form completed by the CRS-trained physician.

The advantages of our study include the fact that most of the study participants had a low educational level, thereby making our results generalizable to the larger population. We developed a solid methodology involving a three-phase validation process, including a qualitative analysis of patient comments. The new form lacks patient-reported BMI which we consider an advantage because of the substantial growing body of evidence supporting inappropriate obesity estimation when BMI calculation is based on patient-reported data. 30 Therefore, medical staff members need to calculate only the BMI and incorporate it to the aggregated score. A few limitations of the study need to be addressed. It was a single-site study, and thus the agreement level in an implementation process needs to be measured. Nonetheless, we achieved a substantial and almost perfect agreement level. Finally, we used American English in this form; therefore, language adaptations may be required for other English-speaking countries.

Patient-reported outcomes are reliable and conveniently obtained measures to assist in clinical practice. For instance, in rheumatology, the routine assessment of patient index data 3 (RAPID3)—which quantifies functional capacity, pain, and global status—appears adequate to document status and monitor the effectiveness of therapies in patients with rheumatoid arthritis; it is also considerably easier to obtain than other physician-reported measurements. 31 Furthermore, this tool has good performance compared with patient-reported measurements in multiple control trials. 32 33 34

The implementation of a formal VTE prevention program, by using a validated risk assessment tool, can effectively reduce VTE occurrence. Using a retrospective database, Catterick and Hunt reported the impact of their national VTE risk assessment tool on secondary care in England. Based on their analysis involving approximately 15 million hospital admissions across England in 2011, the authors estimated that 2,000 secondary diagnoses and 1,200 ninety-day readmissions were avoided. Similarly around 940 deaths owing to VTE were avoided in a population of over 53 million in England in 2011 and 2012. 35 We believe that the availability of a patient-completed CRS will facilitate wider implementation of preoperative risk assessment, which, coupled with risk-based prevention strategies, may decrease the global burden of postoperative VTE. The patient-completed CRS must be carefully checked by a physician to design a specific prophylaxis protocol that balances the risks of thrombosis and bleeding for patients.

## Conclusion

We created and validated a patient-completed CRS form that has an excellent agreement level with the physician-completed form. This study demonstrates that the burden of completing this comprehensive risk assessment is decreased by involving the patients in their care. The average time for the patient to complete the form was 5 minutes, and validated by the physician in a few additional minutes (6 minutes). Further studies are necessary, including those in other popular languages, to assess this methodology to efficiently and accurately complete a patients' risk of VTE.
